# Genetics to Improve Outcomes in Schizophrenia (GENios): A within-case molecular genetic study protocol

**DOI:** 10.1371/journal.pone.0340584

**Published:** 2026-02-05

**Authors:** Sophie E. Smart, Eilidh Fenner, Rhys Humphreys, Amanda Wells, Katherine Fender, Catherine Bresner, Giulia Trauzzi, Isabella R. Willcocks, Sophie E. Legge, Antonio F. Pardiñas, Elliott Rees, Valentina Escott-Price, Peter Holmans, Michael C. O’Donovan, Michael J. Owen, James T. R. Walters

**Affiliations:** 1 Centre for Neuropsychiatric Genetics & Genomics, Division of Psychological Medicine & Clinical Neurosciences, Cardiff University, Cardiff, United Kingdom; 2 Wales, United Kingdom; Public Library of Science, UNITED STATES OF AMERICA

## Abstract

**Introduction:**

Despite significant progress in understanding the genetic basis of schizophrenia, there is a clear gap in our understanding of the genetics of outcomes in schizophrenia, particularly those prioritised by people with the condition. This has impeded progress towards precision psychiatry for schizophrenia and the improvement of outcomes. As genomic cohorts continue to increase in number, size, diversity, and phenotypic detail, sufficient data are now available to enable within-case studies focusing on the genetic basis of outcomes in schizophrenia.

**Methods and analysis:**

The GENios programme of research will use existing and new data to undertake large-scale genomic studies of schizophrenia outcomes. The project focuses on four key outcomes that were prioritised for research by individuals with lived experience of schizophrenia and align with priorities identified in the literature: antipsychotic treatment response; hospital admissions; occupational functioning; and social functioning. The aims of this project are to understand the genetic contributions to these outcomes in schizophrenia to (i) advance current understanding of the biological mechanisms that drive variability in outcomes, (ii) highlight novel drug targets, and (iii) identify genomic predictors of outcomes that can be leveraged for precision medicine.

**Ethics and dissemination:**

The GENios project has been granted ethical approval and collaborators are required to have the appropriate ethical permission in place to contribute data to the project. Findings from this study will be interpreted and disseminated with the involvement of lived experience experts in scientific publications and conferences as well as to wider non-scientific communities.

**Plain English summary:**

Schizophrenia is a severe mental illness that affects how people think, feel, and behave. Whilst current treatments help with some symptoms and effects of the disorder, many people continue to struggle with poor long-term outcomes. Outcomes like repeated hospital stays, difficulties with socialising and relationships, and difficulties with finding and maintaining employment. People with lived experience of schizophrenia have identified some outcomes as research priorities. These research priorities are treatment response, hospital admissions, day-to-day living (e.g. having a job), and having relationships.

Many people with schizophrenia are given antipsychotic medications to treat ‘positive’ symptoms (such as unusual (false or disturbing) thoughts and beliefs, and seeing and hearing things that are not there). These medications work for some people but around 25% to 30% of people with the disorder have symptoms that are not helped by standard antipsychotic treatments. These symptoms are classed as treatment-resistant. Many more people find that antipsychotic medications do not help with ‘negative’ symptoms (such as feeling emotionally blank and having trouble with motivation) and difficulty with cognitive abilities (such as concentrating, planning, and memory).

This project, called GENios, aims to understand why outcomes differ between people with schizophrenia by looking at their genetics. We understand that social factors play an important role in outcomes but this project is specifically focused on genetics. We will analyse data from people with schizophrenia with an aim to uncover the genetic factors that influence outcomes by using data has already been collected by other research projects. We are carrying out this research in order to identify how genes and knowledge of people’s genetics can help develop healthcare that is more tailored to an individual, so-called precision psychiatry. A plain English glossary of terms is included (S1 Table) to help explain technical language used in the main text.

## Introduction

Schizophrenia is a highly heritable disorder, affecting 0.3–0.7% of the population [1,2], with a complex polygenic architecture [3]. It is characterised by disruptions in thoughts, perception, and behaviour, and is heterogeneous in terms of symptoms and outcomes. Despite significant progress in understanding the genetic basis of schizophrenia [3,4], there is a clear and significant gap in our current understanding of the genetics of outcomes in the disorder, which has impeded progress towards precision psychiatry for schizophrenia and the improvement of outcomes [5].

Previous research has estimated that 14% of people with schizophrenia experience persistent improvements in both clinical and social domains (“recovery”) [6] and 16% of people experience symptomatic remission without treatment [7]. With antipsychotic medication, symptom severity can be halved for around 52% of people treated at their first episode of schizophrenia [8] and 37% of people experience symptomatic remission in the long-term [7]. But other studies estimate that only 23% experience a “good” response to non-clozapine antipsychotics [9] and around 23–26% are classified as treatment resistant, meaning their symptoms do not respond to standard antipsychotic treatments [10,11]. In addition, people with schizophrenia have a reduced life expectancy when compared to the population and to people with other severe mental illnesses [12,13]. Many people with schizophrenia have difficulties with social functioning [14,15], including having a partner/a co-habiting relationship [16], finding and maintaining employment [17], fecundity [18], and physical health [19,20]; all of which are thought to contribute to low recovery rates, and to substantial personal, societal, and economic burden [21]. Definitions of what constitutes a “good outcome” for people with schizophrenia vary substantially across studies, time, and domains, and are poorly operationalized [22–25]. Furthermore, they are often not aligned with the self-reported experience of people with schizophrenia [26,27] or their carers [28].

### Genetics of outcomes in schizophrenia

There is some evidence to suggest genetic factors are associated with heterogenous elements of schizophrenia including symptom severity and cognitive impairment [29–33], social cognition [34], functioning [35], hospital admissions [36,37], and treatment resistance [38]. However, these studies have been limited by relatively small sample sizes and/or have considered the impact of genetic factors on individual outcomes, rather than the overlapping genetic architecture of multiple outcomes.

Whilst there is some evidence of an association of higher polygenic risk for schizophrenia with a greater burden of hospitalisations in population-level cohorts [39] and in individuals with schizophrenia [40] and bipolar disorder [41], further research is required to clarify the genetic architecture of outcomes in schizophrenia, and how it overlaps with (and differs from) genetic liability for the disorder itself. As noted in Legge, Santoro [42], studies of treatment-resistant schizophrenia have shown that genetic liability to schizophrenia is inconsistently associated with treatment resistance as an outcome [38,43–47]. Studies focused on the prediction of poor outcomes in schizophrenia suggest that including genetic liability for schizophrenia in prediction models does not substantially improve their performance [48]. In line with these findings, we expect that genetic factors contributing to poor outcomes in schizophrenia may differ from genetic factors contributing to liability for the disorder. A similar pattern (i.e., the genetic basis of liability differing from the genetic basis of disease progression) has been observed in a number of other disorders, including Crohn’s disease [49], Alzheimer’s disease [50], and Parkinson’s disease [51].

Outcomes in schizophrenia are likely to be inter-related, with changes in one domain impacting another [52,53]. Given this observation, we expect that outcomes are likewise genetically correlated, with overlapping genetic architecture, and therefore need to be studied in combination. As such, the underlying biological basis of variation in outcomes remains unknown, and there are no genetic biomarkers that could help isolate targets for outcome-focused therapies or be used to identify outcome groups early in the course of illness. Our poor understanding of the genetic basis of outcomes is, in part, due to the focus of most genetic studies on the presence of a schizophrenia diagnosis in order to identify disease risk genes, rather than a focus on outcomes themselves. As schizophrenia cohorts with genomic data continue to increase in number, scale, diversity, and phenotypic detail, sufficient genomic and phenotypic data are now available to enable within-case studies focusing on the genetic basis of outcomes in schizophrenia.

### Project aims

The GENios programme of research will repurpose data, both existing data from international collaborations and new data generated during the lifetime of the project, to undertake large-scale genomic studies of schizophrenia outcomes.

The project will focus on four key outcome domains that were prioritised for research by those with lived experience of schizophrenia (in the design of this study (see Materials and Methods - PPIE)) and align with research priorities identified in the literature [52,54–57]: antipsychotic treatment response, psychiatric hospital admissions, occupational functioning (e.g., employment), and social functioning (e.g., relationships). The study of genetic factors relating to outcomes in schizophrenia has the potential to advance current understanding of the biological mechanisms that drive variability in outcomes and disease trajectories, aid the development of novel drug targets, and identify genomic predictors which can be exploited to realise the promise of precision psychiatry [58].

#### Biological mechanisms.

Uncovering the overlapping and independent genetic architecture of different outcomes in schizophrenia may help to highlight underlying biological pathways involved in outcomes and thus inform the development of novel outcome-focused therapies. This is an important goal given the limited progress in treatment for schizophrenia since the discovery of antipsychotics, and a lack of effective pharmacological interventions for aspects of the disorder other than psychotic symptoms. Developing interventions that address these unmet needs required a shift towards outcome-driven, rather than diagnosis-driven research. Genetic studies offer a promising route for this, given that drug targets with genetic support are significantly more likely to succeed in clinical trials [59,60].

#### Novel drug targets.

Genetic research focused on outcomes is particularly important given that many treatments across medicine are targeted at modifying disease progression rather than reversing aetiology, and thus genes with a role in progression and outcome are more likely to implicate therapeutic targets than those associated with disease incidence [61].

#### Genomic predictors.

Genomics underpins the two main pillars of precision medicine. First, it offers a route into understanding pathogenesis, outcome and treatment response and the identification of novel therapeutic targets. Second, it can inform risk prediction and patient stratification. Both avenues need to be pursued in parallel to facilitate clinical advances [5,62]. In terms of common variants, polygenic scores specific for outcomes are likely to be necessary for successful patient stratification [42]. This project will also focus on highlighting genetic predictors of poor outcomes, which could facilitate early identification of those who are likely to have poorer outcomes and therefore may need additional healthcare provision; for example, if individuals likely to have TRS could be identified sooner this could be used to support more timely access to clozapine [63]. Evidence suggests interventions have the greatest impacts when implemented early in the course of illness [64,65].

We expect that improving current understanding of the contribution to outcomes of rare genetic variants will be highly valuable for patient stratification. These variants often have large effect sizes in carriers despite modest contributions to population-level metrics such as heritabilities. The high value of accurately assessing rare genetic variation is already evident in other disorders such as cancer [66] and autism spectrum disorder, where rare variants have been shown to be associated with more impairing phenotypes [67,68]. Moreover, studies identifying overlapping rare variants across schizophrenia, autism spectrum disorder, and neurodevelopmental disorders [69–71] suggest that such variants may index schizophrenia cases with a more prominent neurodevelopmental aetiology, who may have a higher likelihood of poor outcomes.

#### Summary of aims.

In summary, the GENios programme of research will undertake large-scale genomic studies of outcomes in schizophrenia using within-case study designs. The aims of this project are to understand the genetic contributions to outcomes prioritised for research by lived experience experts (LEEs), in order to (i) illuminate the biology of poor outcomes, (ii) highlight novel drug targets, and (iii) identify genomic predictors of poor outcomes that can be leveraged for patient stratification and precision medicine. The aim of this Protocol Paper is to provide context and background to the forthcoming studies, and invite further collaborations by outlining the aims, scope, and proposed methodology of the GENios Project.

## Materials and methods

The role of LEEs in the design of this study (Box 1. Pre-Funding PPI Focus Group) and the co-production of this manuscript (Box 1. Manuscript Co-Production) has been reported according to the Guidance for Reporting Involvement of Patients and the Public (GRIPP2) Checklist [72].

### Study design

We will include samples that have (i) genotyping array data and/or exome or whole-genome sequencing data, (ii), phenotype data for at least one of the four key outcomes prioritised for research by LEEs, and (iii) where collaborators have appropriate ethical permission to share the data. To conduct within-case analyses, we will include participants with schizophrenia or schizoaffective disorder. Procedures for research diagnoses vary across cohorts but could be from clinical records (e.g., International Classification of Diseases (ICD) v11 F20 or F25), structured interviews to acquire a research diagnosis, or, when appropriate, self-report [73]. Where data allow us to distinguish between the two main forms of schizoaffective disorder, we will only include those with the depressed subtype as previous work suggests that this is a form of schizophrenia that is modified by an elevated liability to depression [74]. We will not specify exclusion criteria based on time since illness onset, but previous work to harmonize the prospective collection of outcome data recommended collecting outcome data at intervals of six months [54] and we will aim to control for duration of illness in our analyses.

The GENios project will use existing collaborative networks to identify suitable data. Two such networks are the Schizophrenia Working Group of the Psychiatric Genetics Consortium (PGC; secondary analytic proposal approved), which has brought together genotyping data for N = 76,755 people with schizophrenia [3] and the Schizophrenia Exome Sequencing Meta-analysis (SCHEMA) consortium (letter of support provided), which has brought together whole exome sequencing data from more than N = 24,248 people with schizophrenia [4]. Combining data from different settings and countries to address the same research questions provides built-in reproducibility checks under the varying assumptions about how the data were generated, as well as how the results are influenced by national healthcare provision and social structures [75]. This approach is also aligned with UKRI policy, which strongly encourages the reuse of existing data for research [76], and European Union Health Space regulation (Regulation 2025/327) [77].

### Patient and public involvement and engagement (PPIE)

Our approach aims to ensure that stakeholders inform the study at all stages, from design to interpretation, and dissemination (see Box 1 and **Dissemination**).

We plan to establish a stakeholder panel to meet multiple times per year to offer feedback on current project findings and progress, and to shape future research. This panel will consist of 5–10 members, including individuals with schizophrenia, carers, voluntary and community sector representatives, and clinicians. We will aim to make this group as diverse as possible in terms of backgrounds and experiences. The research team will present current findings and project progress at these meetings, and will receive feedback on these, helping shape the research focus and priorities in real-time. To facilitate engagement from our stakeholder panel, we plan to offer training opportunities to support meaningful involvement. These may include training on research methods, genetics, communication skills, and digital technologies.

We will assess the impact of this involvement, in terms of perceived influence in shaping the research and overall experience, by requesting regular feedback from both stakeholders and researchers on the project. We will revise our PPI strategy in response to, and in partnership with, the individuals recruited onto our panel.

**Box 1. Adapted GRIPP2 checklist.**

**Table 1:** Comparison of Kinesiophobia Scores in Tumor Patients With PICC Catheterization With Different Characteristics (*x* ± *s*).

Section and Topic	Pre-Funding PPI Focus Group	Manuscript Co-Production
**1: Aim** Report the aim of PPI in the study	The aim of involving lived experience experts (LEEs) was to gather their opinions about what they would have wanted to know when they first became ill and inform and change the design of the project prior to writing the grant.	The aim was to involve people to co-produce this manuscript. The people involved would provide insights based on their own experience (or that of people they support) that would help inform the design and communication of the GENios project.
**2: Methods** Provide a clear description of the methods used for PPI in the study	Members of the National Centre for Mental Health’s Partnership in Research (PÂR) group, with lived experience of psychosis, were invited to join an online meeting with the five of the authors (JTRW, MOD, SEL, AFP, ER). Participants were paid for their time. RH took part as a participant in this meeting.	We aimed to recruit one or two people with lived experience of schizophrenia, or experience caring for someone with schizophrenia, and advertised the position(s) via the National Centre for Mental Health. SES and EF interviewed people online and selected successful applications based on a pre-determined person specification. SES, EF, RH and AW met online between March and July 2025 to discuss the (i) structure of the paper, (ii) project rationale, (iii) PPI strategy, (iv) methods, (v) introduction, (vi) lay summary, (vii) glossary of terms, (viii) dissemination of findings, (ix) manuscript revisions made by other co-authors. RH and AW were paid for their time, including time reading material in preparation for the meetings and reviewing drafts of the manuscript. In total they each spent approximately 29 hours on this project, at least 12 of which were direct contact with the researchers. RH and AW were given access to Cardiff University training materials on how to read scientific papers and write a scientific manuscript. Questions were always welcome, either during meetings or via email, and SS or EF either provided an immediate response or prepared materials to support upskilling RH and AW in specific areas of psychiatric genetics.
**3: Study results** Outcomes—Report the results of PPI in the study, including both positive and negative outcomes	Our PPI group highlighted the importance of outcomes relating to relationships and employment, as key measures of functioning, as well as service use, particularly hospitalisation, and the need to support those whose symptoms do not respond to standard treatments. Considering outcomes broadly and in combination was seen as valuable based on their own experiences. See: Introduction ≥ Project Aims ≥ Paragraph 2	RH and AW were given access to, and provided comments on, all sections of the manuscript as it was drafted by SES and EF via a shared folder. In meetings they were able to discuss specific aspects of the manuscript and write via dictation. Because of their experience working on this paper and learning more about genetics research, AW has applied to join the Genomics Partnership Wales Patient and Public Sounding Board.
**4: Discussion and conclusions** Outcomes—Comment on the extent to which PPI influenced the study overall. Describe positive and negative effects	As quality of interpersonal relationships was considered of high importance to participants (“She loved me and that’s made the difference looking back”) and so social functioning was included as an outcome domain distinct from occupational functioning. See: Introduction ≥ Project Aims ≥ Paragraph 2	After reviewing the proposed outline of the manuscript, RH and AW moved the PPI strategy subheading to the beginning on the methods section to reflect its role as an important thread throughout the whole project. From the beginning, RH and AW advocated for improving the readability of the manuscript so that it was accessible to more people. While the manuscript was written and edited with this in mind, it was accepted that a manuscript for a scientific journal would not be wholly accessible to those without a scientific background. Therefore, they proposed the inclusion of, and co-wrote, the plain English summary and glossary (S1 Table). See: Plain English Summary See: Plain English Glossary (S1 Table). RH and AW also proposed (i) the PPI strategy for the project and (ii) the plans for dissemination of findings. See: Methods ≥ Patient and Public Involvement and Engagement (PPIE) See: Discussion ≥ Dissemination
**5: Reflections/critical perspective** Comment critically on the study, reflecting on the things that went well and those that did not, so others can learn from this experience	In the interpretation and use of results, participants reflected on the role of stigma and concluded that stigma shouldn’t stop any information being collected from research participants or used in the research. However, they thought that stigma should be a consideration when communicating the research, the results of research, and information that could be clinically informative. Outcomes that are felt to be stigmatising shouldn’t be fed back to participants or patients in a clinical setting. When discussing genetics, participants worried about the risk to their children. The researchers acknowledged these concerns and shared what they know from clinical experience of working in the All Wales Psychiatric Genomic Service; people worry more than they need to about passing psychiatric conditions to their children but when they hear information about what the real risks are, they feel reassured. How risk is communicated impacts people’s wellbeing and needs to be considered carefully when discussing psychiatric genetics research. Finally, there were data privacy concerns which the researchers were worried about (e.g., use of genetic data) that this PPI group were less concerned about. In response to the question, ‘do you consider your genetic data any different from other health data or any other data that is held about you?’, the consensus was that any part of your body can “go wrong” so why would you think about genetic data differently as long as it was anonymised.	There were some institutional barriers that created problems during co-production. Cardiff University no longer supports to use of Zoom Workplace, but Zoom was considered more accessible and user friendly than Microsoft Teams, therefore we used the free version of zoom despite shortcomings such as forced time limits for calls. Cardiff University did not always process payments in a timely manner. RH and AW reflected on their experience of co-producing this manuscript. Their thoughts were grouped into themes by EF and SS and are included below. Safe space “I felt safe and supported to share some difficult experiences, and feel that these were fully taken into account.” “…made me feel welcome as part of the team and showed no stigma or prejudice.” “The most demanding part for the study was the understanding of the jargon involved in some of the reading we had to do, however it was made clear and straightforward that we could ask questions about anything we needed clarification of.” “…professional but always respectful and open to our input and genuinely interested in our lived experiences” Interest in genetic research “I was pleased to learn more about genetics, this is an area that I’ve become more interested in.” “If I was or ever could be in a position to study at university I would consider genetics as a subject of my choice. However as I am still in recovery from my illness I am a long way from considering embarking on a course like that.” Practical support “I greatly appreciated the ease with which additional PA hours were provided for my PA at home to support me in this work.” “The remuneration for my time was greatfully received even if I felt I did not always deserve it.”

### Data analysis plan

**Fig 1:**
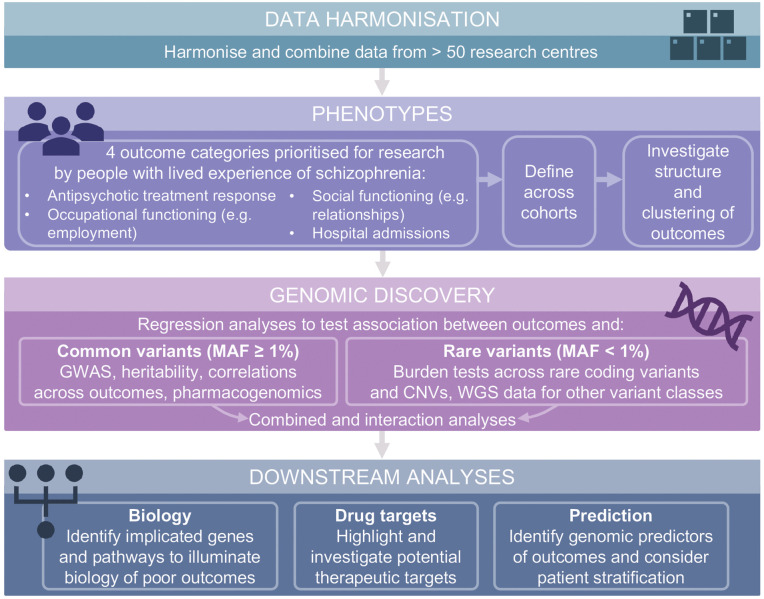
Analytic plan for the GENios programme of research. The overall study workflow, from data harmonisation and phenotype definition to genomic discovery and downstream analyses. MAF = minor allele frequency; GWAS = genome wide association study; CNVs = copy number variants; WGS = whole genome sequencing.

Here we present a broad outline of our analytic plans. Within the lifetime of the project, we anticipate that existing resources and software tools will be updated, and new methods and software will become available. We will therefore use the most appropriate and up-to-date tools available at the time of each analysis. Fig 1 provides an overview of the planned data analysis approach.

#### Data harmonization.

Cohorts will be combined using mega or meta-analysis, whichever is more appropriate given the cohort characteristics and data protection permissions. Although data is expected to be heterogenous, we aim to, where possible, harmonise and pool individual-level data by transforming data to the specifications laid out in a Common Data Model (CDM) and follow existing frameworks [78–80]. To ensure consistency across samples, we will reannotate variants, recalculate allele frequencies, and where necessary re-impute genotype data or recall sequencing variants. Where data cannot be combined, we will still attempt to limit differences in methodology and use cohorts to replicate our analysis and establish external validity.

#### Phenotypes.

The challenge of capturing and amalgamating the breadth of outcome measure data at scale has been a barrier to advancing precision psychiatry research. In addition, the way in which outcomes in schizophrenia have been conceptualised and assessed has changed over time [54,81]. Therefore, for phenotypes, our approach is first to review and visualise raw data, then use the CDM to harmonise existing phenotypes. For pooling individual-level data, a compromise between maximising sample size and loosing granular information is required. Therefore we will harmonise data in levels, retaining the CDM formatted data but also aggregating data to a common denominator or deriving new variables [82]. Depending on the outcome, multiple operational definitions will be available with either large-sample-size/low-granularity or small-sample-size/high-granularity definitions used in sensitivity analysis to support the interpretation of findings.

Additionally, to identify the existence of naturally occurring groups of people with schizophrenia that are phenotypically more homogeneous, we envisage using two approaches: exploratory and confirmatory factor analysis to identify the latent factor structure of outcomes in our data [30,83]; and unsupervised clustering methods (e.g., Agglomerative Hierarchy, K-means, or Density-Based Spatial Clustering of Applications with Noise) to group individuals based on combinations of outcomes [84]. We will test for differences in groupings/factor loadings, using genomic variables, to assess their biological validity [85].

#### Genomic discovery.

We plan to conduct a series of association analyses to identify genetic variants that are associated with our outcomes. Both common (e.g., single nucleotide variants with a minor allele frequency (MAF) ≥ 1%) and rare (e.g., copy number variants (CNVs) and rare coding variants (RCVs) with a MAF < 1%) variants are associated with schizophrenia risk [3,4,86]. We expect the greatest genetic contributions to variance in outcomes will come from common variation, although rare variants are likely to confer much larger effects than common variants in individual carriers, and a sizable contribution may yet come from rare variation that is poorly imputed [87].

***Genome-wide approaches to identify common variants*:** We will conduct within-case genome-wide association studies (GWAS) of each outcome using appropriate association methods. Fixed-effect meta-analysis with inverse-variance weights will be used to pool results across cohorts. We will validate our results using standard post-GWAS methods including trend tests, genetic correlations, and polygenic scoring (PGS) in leave-one-out designs. We will estimate SNV-based heritability.

The focus on multiple outcomes allows the use of genomic discovery approaches that go beyond the standard univariate GWAS framework and exploit correlation and covariance structures between phenotypes. Methodology is developing, but to discover pleiotropic variants associated with multiple outcomes we envisage (i) estimating of the degree of polygenic overlap, allowing for differing directions of effect when these exist, (ii) identifying specific regions that contain pleiotropic variants, which can be used to identify latent factors that indicate shared or distinct polygenicity that can, in turn, be integrated into PGS approaches, and (iii) and the use of cross-phenotype meta-analytic approaches that can detect variants with pleiotropic or antagonistic effects across outcomes. The use of these complementary approaches, each of which exploits different properties of genomic data and phenotypic covariance structures, will allow us to ascertain whether known correlations in outcomes stem from a shared genomic basis.

Genotyping array data can also be used to impute pharmacogenomic variation and metaboliser phenotypes [88]. Antipsychotics are primarily metabolised by CYP2D6, CYP3A4, and CYP1A2 [89] whose activity is strongly genetically influenced, and appreciable proportions of the population are slow or rapid metabolisers [90–92]. Regulatory authorities recognise the clinical relevance of metaboliser phenotypes [93], but in schizophrenia, pharmacokinetic genomic variation has limited impacts on therapeutics [94], drug-gene guidelines from expert consortia are only available for a few antipsychotics [95], and a strong case for clinical utility has only been made for testing for the Duffy-null genotype in individuals of African ancestry [96,97]. We will impute pharmacogenomic variation (“star alleles”) and metaboliser phenotypes based on CYP* genes in order to examine the distribution of enzyme activities and determine the contribution of metaboliser phenotypes to schizophrenia outcomes.

***Genome-wide approaches to identify rare variants*:** We will use genotyping array data to call CNVs and whole exome sequencing data to call RCVs. We will classify alleles by frequency based on our own data and large-scale public sequencing projects (e.g. gnomAD [98]), and group them into protein-truncating variants and missense variants that are predicted to be deleterious according to a number of different classifiers (e.g., MPC [99] and CADD [100]).

We hypothesise that RCVs with the biggest impacts on outcomes will be ultra-rare and highly damaging variants that are concentrated in constrained genes. These variants will therefore be the focus of our initial RCV analyses. We will use metrics such as gnomAD’s loss-of-function observed/expected upper bound fraction (LOUEF) [98] to define constrained genes, but will be alert to methodological developments in this area. We will also explore the associations between outcomes in schizophrenia and RCVs in non-constrained genes, specific gene sets, and in individual genes across the genome. In addition, we will examine alleles with MAF of up to 1%, representing less rare but still uncommon alleles. We will regress outcomes on allelic burden using the latest methods appropriate for the data available. As common and rare variants will be correlated in schizophrenia cases [101], where appropriate, we will correct for common variant effects (via PGS) to prevent spurious associations of rare variants with outcome measures and to enhance power for genomic discovery and prediction.

We anticipate that whole genome sequencing data for approximately 10,000 individuals with schizophrenia will become available during this project. We will apply Illumina DRAGEN pipelines [102] to these data to analyse variants often missed by exome sequencing and genotyping, including structural variants (deletions, insertions, translocations, complex structural variants and tandem duplications larger than 50 base-pairs), short tandem repeats and repeat expansions, and pharmacogenomic alleles that cannot be accurately identified using genotyping array data. We will use the latest methodology to examine whether these classes of rare variants are associated with outcomes in schizophrenia, and integrate them with RCVs to increase power to detect associations [103].

#### Downstream analyses.

The discovery analyses aim to identify and refine individual variants, genes, haplotypes, and sets of genes in which genetic variation is associated with outcomes in schizophrenia. Using these results, we will run downstream analyses designed to achieve the aims of this project. We anticipate the output of these downstream analyses will be linkages between genomic findings and biological annotations, informing hypotheses with respect to the pathophysiology of outcomes and the development of prediction models. Although more speculative, this work has the potential to highlight identification of drugs and other molecules that might improve outcomes.

***Biological mechanisms*:** Biological interpretation can be driven by associations in specific genes, but the expected polygenic nature of the outcomes implies associations are likely to be dispersed across a large number of genes. Therefore, to inform hypotheses about the pathophysiology of these outcomes, we will employ methods to look for enrichment of genetic associations in gene sets representing biological annotations (e.g., tissue expression, single cells, developmental stages, protein interactions). It has been shown that gene set enrichments typically identify the most important aspects of biology for highly polygenic traits [104]. While RCVs precisely pinpoint causal genes, to identify causal common variants that point to molecular mechanisms, GWAS associations will be fine mapped using linkage disequilibrium (LD) or quantitative trait locus (QTL) information integrated with chromatin conformation analysis. As the various sets are not mutually exclusive with respect to gene membership, we will conduct stepwise conditional analyses to resolve which sets are primarily driving the associations [3,105].

As well as seeking unbiased biological insights, we will test hypotheses informed by existing and emerging findings. Firstly, (i) for a given outcome, we hypothesise that common and rare variants will be enriched in the same genes, as is the case for variants associated with schizophrenia risk [3,4]. Secondly, (ii) given that rare variants overlap in schizophrenia, autism spectrum disorder (ASD) and other neurodevelopmental disorders (NDD) [69–71] and in ASD rare variants have consistently been associated with more impairing phenotypes [67,68], we postulate that these variants index schizophrenia cases with more prominent neurodevelopmental aetiology, and predict that NDD genes will be enriched for associations in those with poorer outcomes. Building on this hypothesis, we expect that genes linked to CNS development will be more strongly implicated in poor outcomes compared to genes linked to neuronal function.

***Novel drug targets*:** Improving our understanding of the biological processes underlying poorer outcomes will provide targets for research focusing on developing novel treatments. Rare protective variants can point to targets and therapies that mitigate the effects of disease after its onset [106]. The existence of ‘protective’ variants in schizophrenia is hypothetical, but they need to be considered. One potential example is 22q11 duplication; this has been identified as being depleted in people with schizophrenia [86,107] but not in those with schizophrenia spectrum disorder [108].

We can, in addition, use multi-omics data to provide clues to new treatments. For example, we will develop and test for association enrichment, sets derived from multiple databases representing known drug targets, and genes whose expression is perturbed by drugs in cultured cells [109,110]. We can also attempt to identify drugs whose sets of targets show an excess of protein-protein interactions with significant genes from our association analyses [111]. We will adapt analyses to new or updated resources, such as Open Targets [112].

***Genomic predictors*:** We intend to identify genomic predictors of poor outcomes that can be leveraged for patient stratification and precision medicine. Our aim is to investigate whether and to what extent genomics contributes to prediction modelling, not to arrive at a clinic-ready prediction algorithm, which will require additional work based on our findings for clinical translation. Common variation is likely to be informative in explaining variance in outcomes across people with schizophrenia, while rare variation is more likely to be useful for patient stratification. We will take various approaches to identify novel strata within cases. Improvements in PGS-based prediction are likely to be obtained by methodological developments, and we will therefore evaluate our predictors against newly emerging methods and adopt more powerful approaches should they become available. We will report all our analysis to identify predictors in accordance with the Transparent Reporting of a multivariable prediction model for Individual Prognosis Or Diagnosis (TRIPOD) statement [113].

There is evidence that phenotypic expression in one disorder is modified by liability to other traits [30,74,114–116]. For example, the schizophrenia PGS is associated with schizophrenia as an outcome in undifferentiated first episode psychosis [117], with poor responses to lithium in bipolar disorder [118] and to antidepressant treatment in major depression [119,120]. Therefore, we will explore whether there is any evidence for cross-outcome genomic prediction in our cohorts. The rationale for cross-trait genomic prediction is supported by extensive pleiotropy [121], dimensional models of psychopathology, and genetic correlation. For example, TRS is genetically correlated with liability to cognition but not to schizophrenia per se [38] and PGS representing fractions of liability derived from a joint analysis of schizophrenia, bipolar disorder, and major depressive disorder differentially predict clinical features in people with bipolar disorder [122]. Therefore, we will test the predictive value of PGS derived from studies of other psychiatric disorders, as well as cognitive, personality and neurodevelopmental traits. We will also test PGS representing fractions of liability that are shared between, or that are relatively specific to, disorders/traits with well-powered GWAS that are correlated with schizophrenia (bipolar disorder, major depressive disorder, and IQ) and the outcomes. Each PGS will be tested individually for relevance to outcome prediction. Those that are informative will be tested jointly [123,124] in multivariable analysis to identify those with independent contributions, and to quantify their combined value for predicting outcomes.

CNVs and RCVs are likely to have limited impacts on outcomes at a population level, but they may offer the most rapid gains for precision medicine in schizophrenia as they can confer large individual effects (ORs 2–70) [4,71,86]. These variants have the potential to define cases with shared pathogenesis who might benefit from the same targeted treatments [125]. We will take a gene-first approach seeking novel case strata with specific molecular diagnoses based on RCVs or CNVs, with each putative stratum being based on a single associated gene. We will then review in detail all sources of data, seeking commonality at all phenotypic levels, as groups may represent novel syndromes suitable for targeted treatments. We acknowledge this is exploratory and that such syndromes may not exist, or that there will be insufficient informative individuals with relevant variation in a single gene.

Seeking to maximise genomic prediction and/or stratification, we will conduct joint analyses of rare and common genomic variation, including pharmacogenomic variants [67,126]. People with schizophrenia who carry rare variants have relatively low common variant burdens [127], suggesting that predictions and stratification based only on common variants may be misleading for rare variant carriers; as recently demonstrated for metabolic traits [128]. The effects of common variants, CNVs, RCVs (and, if relevant, other classes of rare variants identified from whole genome sequencing studies) in genes and associated gene sets will be included together in our prediction models. A simple approach is to include rare variant risk scores and PGS in a multivariable regression (as has been done in studies of autism spectrum disorder [67]. In these models, rare variant risk would be represented by the number of qualifying variants (i.e., variants of classes associated with outcomes) carried by each individual, but we will determine if performance is improved by weighting variants (e.g., by effect size, or aggregate effect sizes for variants of their class (mutation type, functional pathway etc.)). The optimal approach to combine common and rare variants is dependent on the modes of co-action of these variants, therefore we will explore regression models that allow for additive, multiplicative and non-linear effects, as has been previously used for Alzheimer’s disease [129]. The discriminative ability of the models will be quantified using the area under the receiver operating characteristic curve for binary outcomes and the proportion of variance explained for quantitative outcomes.

#### Ancestry.

We will include individuals from multiple ancestries, identified using biogeographic inference [96,130], in our analyses. Where a cross-ancestry analysis is not possible (which may be the case for rare variant analyses if sample sizes across ancestries are limited), we will run within-ancestry analyses ensuring all participants are included, and then meta-analyse findings across ancestries. The multi-ancestry nature of our cohorts will also enable us to explicitly test the transferability of genomic prediction models across diverse ancestries and evaluate their potential algorithmic biases [131].

#### Sex.

Sex is a critical determinant of health that can influence risk, presentation, treatment response, and outcomes of a disorder [132], including for people with schizophrenia [133] where male sex is associated with poorer outcomes [134,135]. Sex refers to the biological attributes that differentiate female and male individuals and is different to gender which refers to an aspect of a person’s identity [136]. The mechanisms underlying sex differences in schizophrenia are unclear but are likely to involve genetic factors, biological, cognitive, and social influences. Across psychiatric disorders, work is attempting to estimate how genetic effects differ between males and females and whether the source of genetic variation is different [137,138]. Although the GENios project will not be able to differentiate between gender-related factors and hormone levels [139], we will report sex-disaggregated analysis as recommended by the MESSAGE framework [140], which will be particularly important for making inferences about personalised medicine [141].

#### Power analyses.

Most of the outcomes have not been consistently defined or studied in combination with genetic variants, and it is therefore difficult to estimate: the rates of outcomes in people with schizophrenia; the proportion of people who carry rare variants; and effect sizes. Of the four outcome domains, there is only literature to accurately estimate power for our analysis of antipsychotic treatment response, and specifically common variant discovery and treatment-resistance. In 100,000 schizophrenia cases, of whom 26% are treatment resistant, there is > 80% power to detect a variant accounting for 0.07% of liability at genome-wide significance (p = 5 x 10^−8^) [142].

### Data management plan

All study data will be managed in compliance with relevant institutional policies and data protection regulations. Genomic and clinical datasets will be stored on secure servers with appropriate backup and access controls. Data processing will follow established best-practice pipelines, and metadata will adhere to recognised standards to ensure reproducibility. Pseudo-anonymised data will be shared through approved repositories under controlled access, with encrypted transfers and governance by authorised committees. All analysts will comply with confidentiality agreements and ethical approval requirements.

### Ethics

The GENios project has been granted ethical permission by Cardiff University’s School of Medicine Research Ethics Committee (SMREC Reference Number: 19/72). The harmonised and aggregated dataset used in this study will be compiled from multiple international contributing cohorts. Each of these cohorts confirmed they obtained ethical approval and followed their own procedures for written informed consent in accordance with local regulations at the point of data collection.

Our research team first received anonymised datasets from collaborators in late 2024, and additional datasets have been collected on a rolling basis, which will continue until the end of 2026. Only fully anonymised data are provided to our research team.

### Timeline

The first stage of the project involves collaboration formation (2024–2025), then data co-ordination, curation, and harmonisation (2024–2026). We are currently in the process of data co-ordination (the collection and integration of existing data from international collaborators) and expect to complete this process by the end of 2026.

As of December 2025, we have received access to data for approximately 30,000 participants and will begin harmonising these data in early 2026. We expect harmonisation of the full cohort to be completed by March 2027. The second stage of the project will focus on genomic discovery (2026–2027), and the third stage on downstream analyses (2027–2028). We expect to have interim genomic discovery results by December 2026, full genomic discovery results in December 2027, and downstream analysis results by October 2028.

## Discussion

### Dissemination

Findings from this study will be interpreted and disseminated with the involvement of LEEs. As the study progresses, we will maintain regular engagement with collaborators through project update meetings, during which preliminary findings will be shared and discussed. Results will be disseminated to the scientific community through peer-reviewed publications and presentations at international conferences. We will also disseminate our findings to research funders in order to shape research funding strategy, for example, through knowledge-exchange with existing funding schemes and ongoing projects that we are part of, including the EU-funded projects REALMENT [143] and Psych-STRATA, the Wellcome-funded GlobalMinds project, and the UKRI-funded Mental Health Platform Brain and Genomics Hub. We will use our findings to build partnerships with industry (including health services) that will inform improvements in care and lead to better classification and treatment. To ensure the accessibility of our findings beyond academic audiences, we plan to produce plain English summaries of our research and the key findings. These summaries will be co-developed with LEEs (as in this manuscript – see ‘Plain English Summary’) and made available in accessible formats. Digital versions will be disseminated online (via a website and/or social media channels), and physical copies will be distributed through trusted local channels (e.g., care homes and supported accommodation, mental health groups/teams, community organisations, local pharmacies). We will provide a way for readers to reflect on the findings and share their views with the study team. Additionally, we will seek opportunities to include short updates on project findings on our website and in newsletters circulated by local community groups and organisations. This will further disseminate findings to broad and diverse stakeholder communities.

### Limitations

Delays to data delivery may lead to a reduction in sample size and consequently power. To mitigate this risk, we have undertaken substantial groundwork to engage collaborators from across the two major schizophrenia genomics consortia (PGC and SCHEMA) and identified new samples. We have confirmed the availability and coverage of relevant phenotypic data in each dataset and have obtained agreement to access it. Given that we are repurposing data from multiple cohorts, originally collected for other purposes, a major challenge will be harmonising phenotypes across datasets. Addressing this challenge will require expert guidance from lived experience experts and our scientific collaborators, as well as data-driven approaches. In addition, we cannot know a priori which classes of genetic variants are most important for the assessed outcomes, but our genome-wide focus on all types of currently assayable genomic variation makes our experimental design robust to heterogeneity in genetic architecture across phenotypes.

Despite these challenges, the broad scope and potential translational impact of the GENios programme of research, combined with strong collaborative and lived experience involvement, positions this project to make a meaningful contribution to understanding the genetic basis of outcomes in schizophrenia. By integrating diverse genomic data and harmonised phenotypes from across cohorts, this study will support progress towards more personalised approaches to treatment and care for the disorder.

## Supporting information

S1 Table Plain English glossary of terms. (DOCX)
