# Supplementary material for: Genetics to Improve Outcomes in Schizophrenia (GENios): A within-case molecular genetic study protocol
Source: PLoS One. 2026 Feb 5;21(2):e0340584. doi: 10.1371/journal.pone.0340584 (PMC12875459; doi:10.1371/journal.pone.0340584)
Supplement: S1 Table — (DOCX) [file pone.0340584.s001.docx]

**S1 Table.** **Plain English Glossary of Terms.**

| **Term** | **Definition** |
| --- | --- |
| Aetiology | The cause or causes of an illness. |
| Alleles | DNA patterns are formed from DNA letters that come in pairs. We call each letter in a pair an allele. |
| Biomarkers | Biological signs (e.g. protein levels) that can be measured (e.g. through a blood test) that tell us something about a person’s health |
| Burden test | Testing whether there is an association between the number of variants within a specific gene (or set of genes) and an outcome. |
| Cognition | The mental processes that take place in the brain including thinking, attention, learning, and understanding things. |
| Common variants | Genetic variants that are present in the population at frequencies of 1% or more. |
| Copy number variant (CNV) | Genetic variants for which the number of copies of a specific section of DNA varies between individuals (e.g. sections which are missing or that are repeated). |
| Fecundity | A measure of the number of children an individual has. People with psychiatric disorders, notably schizophrenia, tend to have fewer children than the general population average. This is likely because of social reasons rather than biology. |
| Genetic Correlation | An estimate of the average genetic similarity between two outcomes. For example, if the same gene differences appeared in two or more of our outcomes, then they would have a high genetic correlation. |
| Genome-wide association study (GWAS) | A type of study that looks across the genome to find genetic differences that are linked to an outcome. |
| Genotype data | Genetic information from specific spots in DNA patterns where people commonly differ. |
| Harmonisation | Making things comparable |
| Heterogeneous | Differences across people. Schizophrenia is heterogenous in that it is not the same for everyone; symptoms and treatment response can vary widely. |
| Liability | The total amount of risk someone carries for developing a condition or outcome, based on their genes and environment. |
| Minor Allele Frequency (MAF) | Where a genetic variant exists in at least two forms, each form is called an allele. The least common allele is called the minor allele, and the minor allele frequency is how often this minor allele occurs in the population. |
| Negative symptoms of schizophrenia | Symptoms that involve a loss of or lack of something. These include things like reduced emotional expression or social withdrawal, and cognitive difficulties, such as concentrating, planning, and memory. In schizophrenia, negative symptoms are harder to treat than positive symptoms. |
| Novel drug target | A drug target is a specific part of the body that a drug interacts with to produce a healing effect. “Novel” means “new”. It means these targets are not currently being used by existing drugs. |
| Pathogenesis | The development and progression of a disorder, including how it begins and how it develops and progresses. |
| Pharmacogenomic variation | Differences in a person’s DNA that impact how they respond to medications (e.g. how well a drug works for someone, or what dose they might need) |
| Pharmacological | Relates to pharmacology, which is the science of medicines and drugs, and how they affect the body. |
| Pleiotropy | When a genetic variant or gene influences more than one outcome. |
| Polygenic architecture | An outcome would have a polygenic architecture if many different genetic variants from across all of your DNA contribute to that outcome. |
| Polygenic Score (PGS) | Adding up how many different genetic variants (that may affect an outcome) an individual has. |
| Positive symptoms of schizophrenia | Symptoms that involve the addition of something, for example hallucinations or delusions. |
| Precision psychiatry | Tailoring psychiatry treatment to an individual (e.g. based on their genetics) |
| Prevalence | How common a condition is in a group of people at a certain time. For example, if 1 in 100 people have a disorder, its prevalence would be 1%. |
| Rare variants | Genetic variants that are present in the population at frequencies of  less than 1%. |
| Regression analysis | The word regression here is a use referring to a statistical method (see definition). Regression analysis means looking for relationships between different things (e.g. genetic factors and an outcome), often to see if one thing predicts another. |
| Sequencing data | Genetic information that tells us the exact pattern of DNA letters (the genome). This can involve the whole genome or part of it. |
| Statistical methods | A type of maths that’s used to analyse information. Statistical methods are often used in research. |
| Stratification | Grouping people based on shared characteristics (e.g. symptom type, genetics, or antipsychotic treatment response) with an aim to treat each group more effectively. |
| Treatment resistant schizophrenia (TRS) | Schizophrenia in which symptoms don’t respond to at least two different antipsychotic medications. Clozapine can then be prescribed. |
| Within-case studies | A study that only involves people with a particular diagnosis (“cases”) and no people without that diagnosis (“controls”). |
